# Flat edge modes of graphene and of *Z*_2 _topological insulator

**DOI:** 10.1186/1556-276X-6-358

**Published:** 2011-04-21

**Authors:** Ken-Ichiro Imura, Shijun Mao, Ai Yamakage, Yoshio Kuramoto

**Affiliations:** 1 Department of Quantum Matter, AdSM, Hiroshima University, Higashi-Hiroshima, 739-8530, Japan; 2 Department of Physics, Tohoku University, Sendai, 980-8578, Japan; 3 Department of Physics, Tsinghua University, Beijing, 100084, PR China

## Abstract

A graphene nano-ribbon in the zigzag edge geometry exhibits a specific type of gapless edge modes with a partly flat band dispersion. We argue that the appearance of such edge modes are naturally understood by regarding graphene as the gapless limit of a *Z*_2 _topological insulator. To illustrate this idea, we consider both Kane-Mele (graphene-based) and Bernevig-Hughes-Zhang models: the latter is proposed for HgTe/CdTe 2D quantum well. Much focus is on the role of valley degrees of freedom, especially, on how they are projected onto and determine the 1D edge spectrum in different edge geometries.

## Introduction

Graphene has a unique band structure with two Dirac points, *K*- and *K'*-valleys--in the first Brillouin zone [1,2]. Its transport characteristics are determined by the interplay of such effective "relativistic" band dispersion and the existence of valleys [3,4]. The former induces a "Berry phase *π*," manifesting as the absence of backward scattering [5]. A direct consequence of this is the perfect transmission in a graphene *pn*-junction, or Klein tunneling [6,7], whereas its strong tendency not to localize, i.e., the anti-localization [8-10], is also a clear manifestation of the Berry phase *π *in the interference of electronic wave functions. Another feature characterizing the electronic property of graphene lies in the appearance of partly *flat band *edge modes in a ribbon geometry [11-13]. It has been proposed that such flat band edge modes can induce nano-magnetism. The flat band edge modes also show robustness against disorder [14]. The Dirac nature in the electronic properties of graphene is much related to the concept of *Z*_2 _topological insulator (*Z*_2_TI). A *Z*_2_TI is known to possess a pair of gapless *helical *edge modes protected by time reversal symmetry. Similar to the gapless *chiral *edge mode of quantum Hall systems, responsible for the quantization of (charge) Hall conductance [15], the *helical *edge modes ensure the quantization of spin Hall conductance. The Kane-Mele model [16,17] (= graphene + *topological *mass term, induced by an intrinsic spin-orbit coupling) is a prototype of such *Z*_2_TI constructed on a honey-comb lattice. Edge modes of graphene and of the Kane-Mele model show contrasting behaviors in the zigzag and armchair ribbon geometries [4,11]. In this article, we argue that the flat band edge modes of zigzag graphene nano-ribbon can be naturally understood from the viewpoint of underlying *Z*_2 _topological order in the Kane-Mele model. To illustrate this idea and clarify the role of valleys, we deal with the Kane-Mele and the Bernevig-Hughes-Zhang (BHZ) models [18] in parallel, the latter being proposed for HgTe/CdTe 2D quantum well [19].

## Flat band edge modes in garphene and Kane-Mele model for *Z*_2 _topological insulator

Let us consider a minimal tight-binding model for graphene: , where *t*_1 _is the strength of hopping between nearest-neighbor (NN) sites, *i *and *j*, on the hexagonal lattice. The tight-binding Hamiltonian *H*_1 _has two gap closing points,  and , in the first Brillouin zone. In the Kane-Mele model [16], hopping between next NN (NNN) sites (hopping in the same sub-lattice) is added to *H*_1_, the former being also purely imaginary: , where 〈〈...〉〉 represents a summation over NNN sites. *s_z _*is the *z*-component of Pauli matrices associated with the real spin, and *ν_ij _*is a sign factor introduced in [16]. The origin of this NNN imaginary hopping is intrinsic spin-orbit coupling consistent with symmetry requirements. *it*_2 _induces a mass gap of size  in the vicinity of  and .

**Figure 1:**
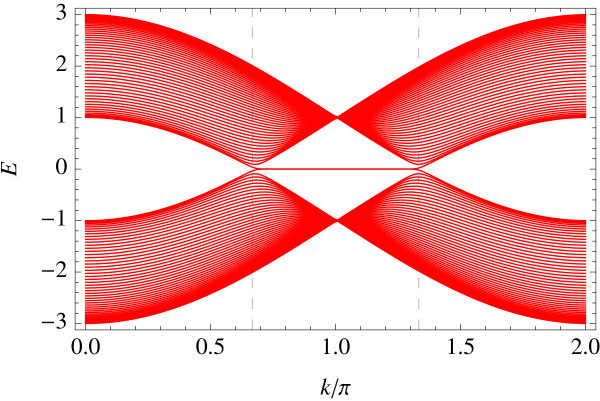
**Zigzag edge modes of graphene**.

**Figure 2:**
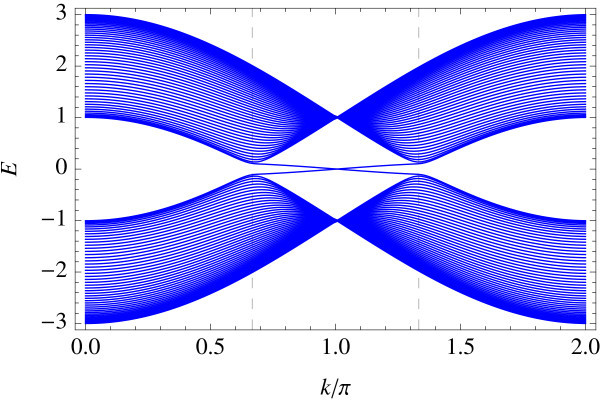
**Zigzag edge modes of the Kane-Mele model**.

Tight-binding implementation allows for giving a precise meaning to two representative edge geometries on hexagonal lattice: armchair and zigzag edges (a general edge geometry is a mixture of the two). Different geometries correspond to different ways of projecting the bulk band structure to 1D edge axis. In the armchair edge, the two Dirac points  and  reduce to an equivalent point whereas in the zigzag edge, they are projected onto inequivalent points on the edge, i.e., . Figures 1 and 2 show the energy spectrum of graphene (Figure 1) and of the Kane-Mele model (Figure 2) in the zigzag ribbon geometry. *t*_2_/*t*_1 _ratios are chosen as *t*_2_/*t*_1 _= 0 and *t*_2_/*t*_1 _= 0.05 in the above two cases, respectively (*t*_1 _is fixed at unity). Dotted lines represent projection of  and . In the Kane-Mele model (with a finite *t*_2_) the existence of a pair of gapless helical edge modes is ensured by bulk-edge correspondence [20].

Therefore, they appear both in armchair and zigzag edges. In the graphene limit: *t*_2 _→ 0, however, the edge modes survive only in the zigzag edge geometry, as a result of different ways in which - and -points are projected onto the edge. In the armchair edge, the helical modes at finite *t*_2 _are absorbed in the bulk Dirac spectrum in this limit. In the zigzag edge, on the contrary, the helical modes connecting  and  survive but become completely flat in the limit *t*_2 _→ 0. Notice that  and  interchange under a time-reversal operation. In the sense stated above, we propose that the flat band edge modes of a zigzag graphene ribbon is a precursor of helical edge modes characterizing the *Z*_2 _topological insulator.

Note here that such surface phenomena as fiat and helical edge states are characteristics of a system of a finite size, and the evolution of such gapless surface states is continuous, free from discontinuities characterizing a conventional phase transition as described by the Landau theory of symmetry breaking. The study of a system of a finite size *L *can be employed to determine the presence (or absence) of a topological gap with the precision of 1/*L*. The behavior of such gapless surface states that exist on the topologically non-trivial side is continuous, up to and at the gap closing. They also evolve continuously into gapped surface states on the trivial side. The flat edge modes appear at the gap closing when they do.

## Edge modes of BHZ model on square lattice

Inspired by the contrasting behaviors of edge modes of graphene and of the Kane-Mele model in the zigzag and armchair ribbon geometries, let us consider here the BHZ model in different edge geometries [21]. The BHZ model in the continuum limit is a low-energy effective Hamiltonian describing the vicinity of a gap closing at Γ = (0, 0) of the 2D HgTe/CdTe quantum well. It can be also regularized on a 2D square lattice in the following tight-binding form:(1)

where Γ*_x _*and Γ*_y _*are 2 × 2 hopping matrices:(2)

***σ ***= (*σ_x_*, *σ_y_*, *σ_z_*) is another set of Pauli matrices different from ***s ***= (*s_x_*, *s_y_*, *s_z_*), and represents an orbital pseudo spin. Note also that Equation 1 describes only the (real) spin up part. To find the total time-reversal symmetric Hamiltonian, Equation 1 must be compensated by its Kramers partner [18]. The lattice version of BHZ model acquires four gap-closing points shown in Table 1, if one allows the original mass parameter Δ to vary beyond the vicinity of Δ = 0. The new gap closing occurs at different points in the Brillouin zone from the original Dirac cone (Γ-point), namely, at *X*_1 _= (*π*/*a*,0), *X*_2 _= (0, *π*/*a*) and *M *= (*π*/*a*, *π*/*a*). The gap closing at *M *occurs at Δ = 8*B*, whereas the gap closings at *X*_1 _and *X*_2 _occur simultaneously when Δ = 4*B*. Thus, at Δ = 4*B*, "valley" degrees of freedom appear as a consequence of the square lattice regularization. In contrast to *K*- and *K'*-valleys in graphene; however, the valleys here, *X*_1 _and *X*_2_, are two of the four time-reversal invariant momenta in the 2D Brillouin zone. Away from the gap closing, the spectrum acquires a mass gap. The sign of such a mass gap, together with the chirality *χ*, determines their contribution to the spin Hall conductance: . Here, ↑ and ↓ refer to the real spin. In the table, only the sign in front of *e*^2^/*h *is shown. The symmetry of the valence orbital is indicated in the parentheses, which is either, *s *(normal gap) or *p *(inverted gap), corresponding, respectively, to the parity eigenvalue: *δ_s _*= +1 or *δ_p _*= -1. The latter is related to the *Z*_2 _index *ν *as (-1)^ν ^= ∏*_DP _δ_DP _*[22]. The *Z*_2 _non-trivial phase is characterized by *ν *= 1, and corresponds to the range of parameters: 0 < Δ/*B *< 4 and 4 < Δ/*B *< 8. Note that in the *ν *= 1 phase, contributions from Γ and *M *to  cancel, whereas those from *X*_1 _and *X*_2 _reinforce each other. In this sense, the role of *X*_1 _and *X*_2 _are analpogous to that of *K *and *K' *in the *Z*_2 _non-trivial phase of the Kane-Mele model.

**Table 1:** Four Dirac cones of BHZ model on square lattice

Dirac Points (DP)	Γ	*X*_1_	*X*_2_	*M*		∏_DP_*δ*_DP_
= (*k_x_, k_y_*) at the DP	(0, 0)	(0, *π*/*a*)	(*π*/*a*, 0)	(*π*/*a*, *π*/*a*)		

Mass gap	Δ	Δ - 4*B*	Δ - 4*B*	Δ - 8*B*		

Chirality χ	+	-	-	+		

Δ < 0	- (*p*)	+ (*s*)	+ (*s*)	- (*p*)	0	+1
0 < Δ < 4*B*	+ (*s*)	+ (*s*)	+ (*s*)	- (*p*)	2*e*^2^/*h*	-1
4*B *< Δ < 8*B*	+ (*s*)	- (*p*)	- (*p*)	- (*p*)	-2*e*^2^/*h*	-1
8*B *< Δ	+ (*s*)	- (*p*)	- (*p*)	+ (*s*)	0	+1

**Figure 3:**
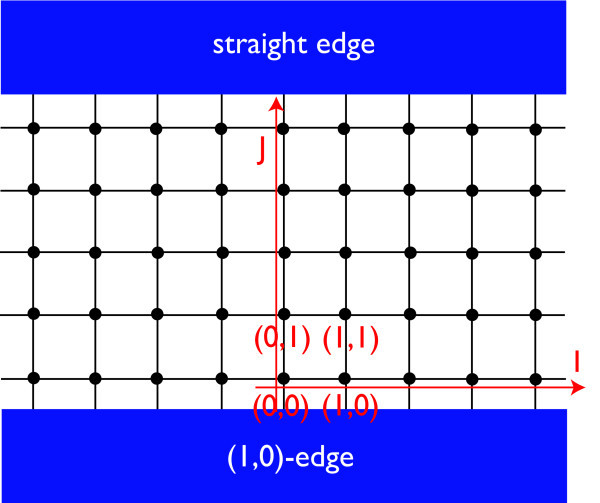
**Straight edge geometry**.

**Figure 4:**
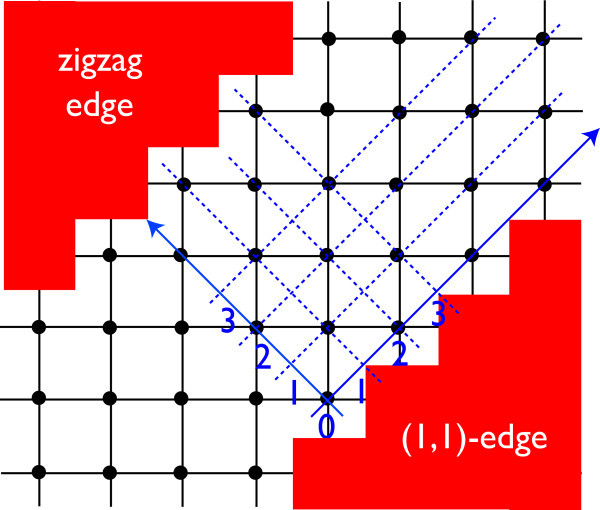
**Zigzag edge geometry**.

Figures 3 and 4 shows two representative edge geometries on a 2D square lattice: straight (Figure 3) vs. zigzag (Figure 4) edge geometries. In analogy to the projection of *K*- and *K'*-points onto the edge in armchair and zigzag edge geometries, notice that here in the straight edge, Γ and *X*_2 _are superposed on the *k_x_*-axis. Similarly, *X*_1 _and *M *are projected onto the same point. In the zigzag edge of BHZ model, Γ and *M *are superposed, whereas *X*_1 _and *X*_2 _reduce to an equivalent point at the zone boundary.

### Straight edge

**Figure 5:**
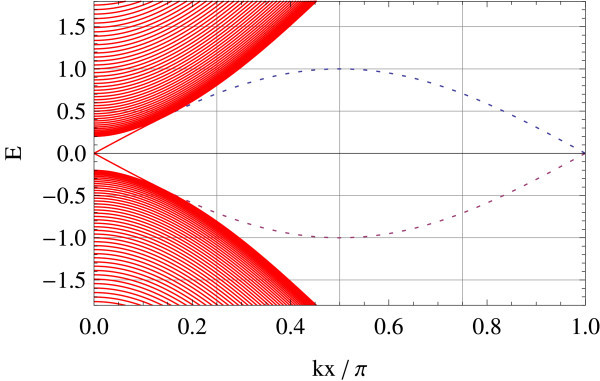
**Energy spectrum of BHZ model: straight edge, Δ/*B *= 0.2**.

**Figure 6:**
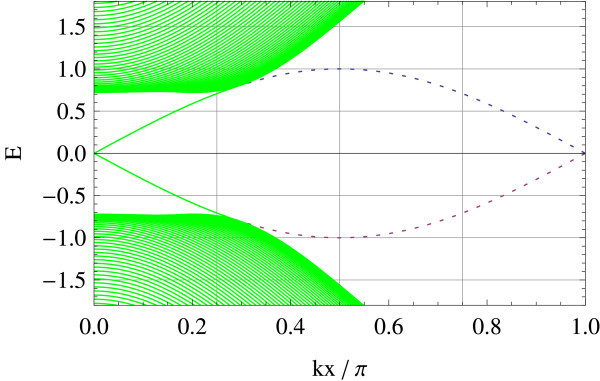
**Δ/*B *= 0.8**.

**Figure 7:**
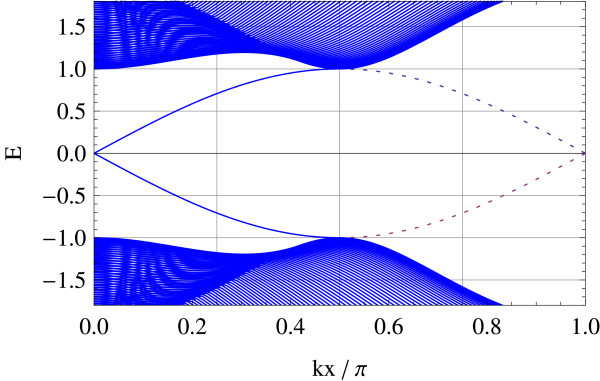
**Δ/*B *= 2**.

**Figure 8:**
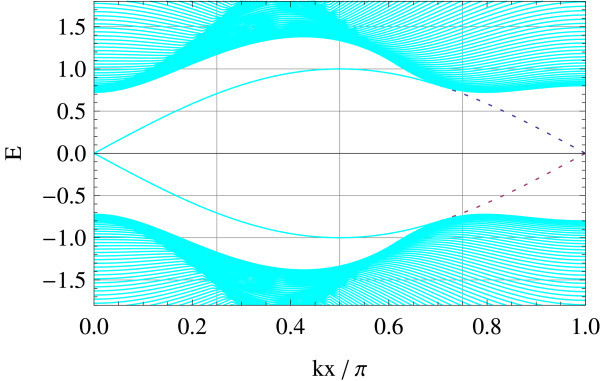
**Δ/*B *= 3.2**.

**Figure 9:**
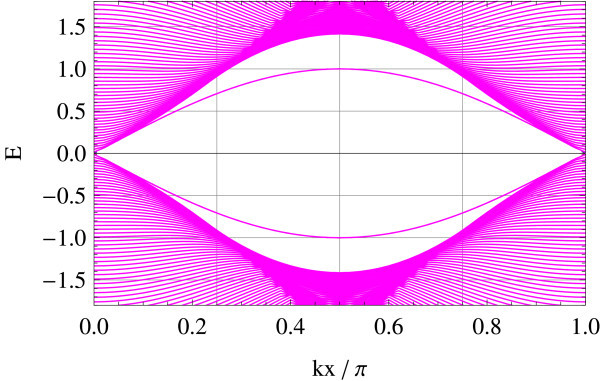
**Δ/*B *= 4**.

**Figure 10:**
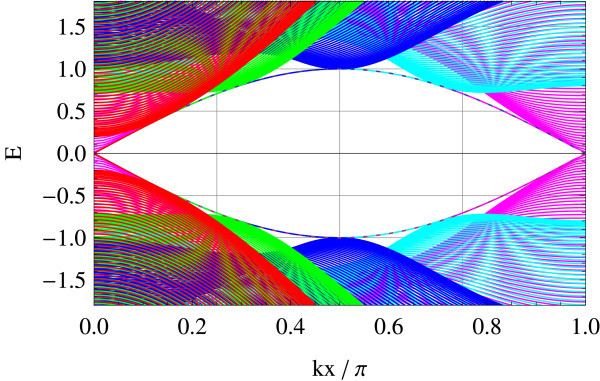
**Comparison of Figures 5-10**.

The edge spectrum in the straight edge geometry is obtained analytically as [21,23], *E*(*k_x_*) = ± *A *sin *k_x_*. As is clear from the expression, the spectrum does not depend on Δ/*B*, which is very peculiar to the straight edge case. Only the range of the existence of edge modes changes as a function of Δ/*B *(see Figures 5, 6, 7, 8, 9 and 10) [21]. In the figure, the energy spectrum (of edge + bulk modes) obtained numerically for a system of 100 rows is shown in a ribbon geometry with two straight edges. Starting with Figure 5 (spectrum shown in red), the value of Δ/*B *is varied as Δ/*B *= 0.2, Δ/*B *= 0.8 (green, Figure 6), Δ/*B *= 2 (blue, Figure 7), Δ/*B *= 3.2 (cyan, Figure 8), and Δ/*B *= 4 (magenta, Figure 9). All of these five plots are superposed in the last panel (Figure 10). *A *and *B *are fixed at unity. The dotted curve is a reference showing the exact edge spectrum. The plots show explicitly that the edge spectrum at different values of Δ/*B *are indeed on the same sinusoidal curve.

### Zigzag edge

In contrast to the straight edge case, deriving an analytic expression for the edge spectrum in the zigzag edge geometry is a much harder task [24].

**Figure 11:**
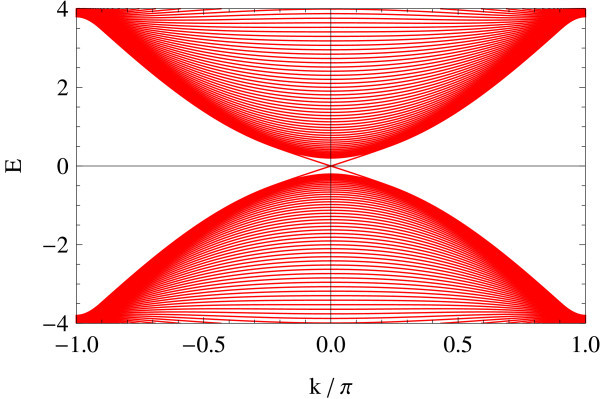
**Energy spectrum of BHZ model: zigzag edge, Δ/*B *= 0.2**.

**Figure 12:**
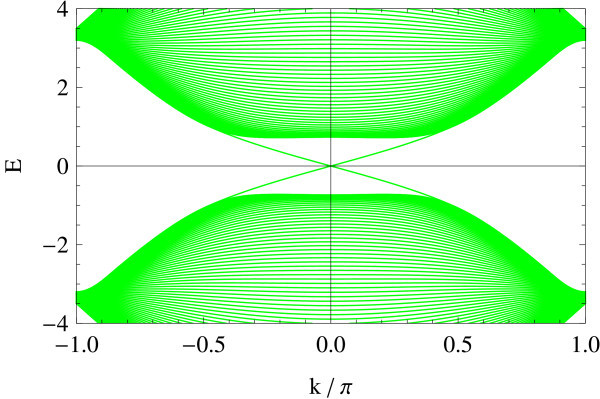
**Δ/*B *= 0.8**.

**Figure 13:**
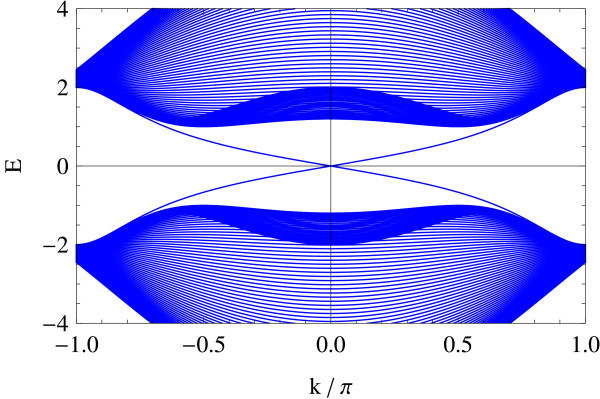
**Δ/*B *= 2**.

**Figure 14:**
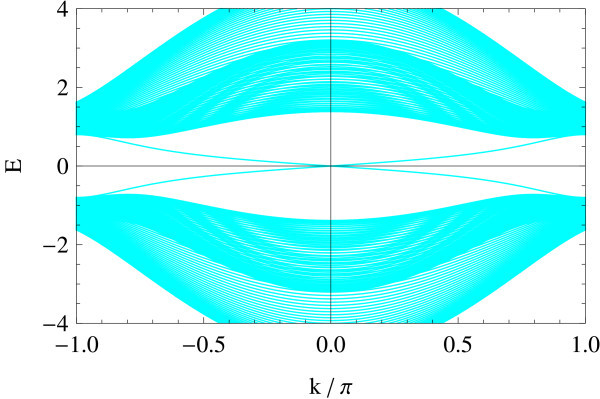
**Δ/*B *= 3.2**.

**Figure 15:**
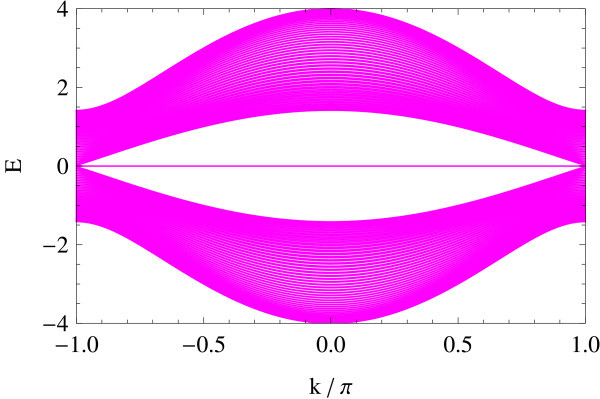
**Δ/*B *= 4**.

**Figure 16:**
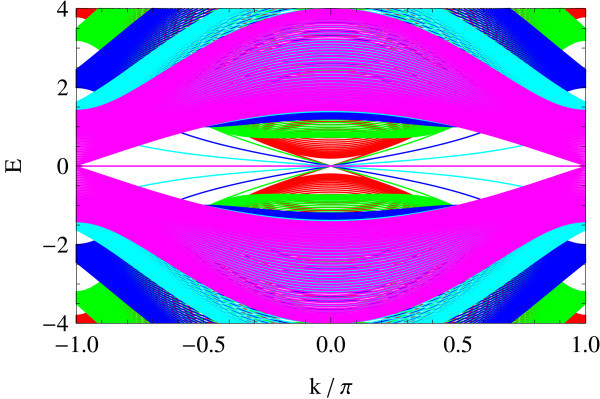
**Comparison of Figures 11-15**.

The edge spectrum has also a very different character from the straight edge case; typically, its slope in the vicinity of crossing points varies as a function of Δ/*B *(see Figures 11, 12, 13, 14, 15 and 16): Δ/*B *= 0.2 (red, Figure 11), Δ/*B *= 0.8 (green, Figure 12), Δ/*B *= 2 (blue, Figure 13). Δ/*B *= 3.2 (cyan, Figure 14), and Δ/*B *= 4 (magenta, Figure 15). These five plots are superposed in the last panel (Figure 16) to show that the edge spectra at different values of Δ/*B *are, in contrast to the straight edge case, *not *on the same curve. Even in the long-wave-length limit: *k *→ 0, their slopes still differ.

At Δ/*B *= 4, the edge spectrum becomes completely flat and covers the entire Brillouin zone. Notice that the horizontal axis is suppressed to make the edge modes legible. Analogous to the flat edge modes in graphene, these edge modes connect the two valleys *X*_1 _and *X*_2 _in the bulk, though they reduce to an equivalent point on the edge. As the bulk spectrum is also gapless at Δ/*B *= 4, the flat edge modes indeed touch the bulk continuum at the zone boundary.

## Conclusions

We have studied the edge modes of graphene and of related topological insulator models in 2D. Much focus has been on the comparison between the single versus double valley systems (Kane-Mele versus BHZ). We have seen that a flat edge spectrum appears in the two cases, whereas in the latter case, the flat band edge modes connect the two valleys that have emerged because of the (square) lattice regularization. The appearance of flat band edge modes in the zigzag graphene nano-ribbon was naturally understood from such a point of view.

## Abbreviations

BHZ: Bernevig-Hughes-Zhang.

## Competing interests

The authors declare that they have no competing interests. 

## Authors' Contributions

KI carried out much of the analytical and numerical studies, and wrote the manuscript. SM made a significant contribution to the analytic part. AY contributed mainly to the numerical part. YK supervised the project.
